# Development of lateral flow assays to detect host proteins in cattle for improved diagnosis of bovine tuberculosis

**DOI:** 10.3389/fvets.2023.1193332

**Published:** 2023-08-15

**Authors:** Hamza Khalid, Louise Pierneef, Anouk van Hooij, Zijie Zhou, Danielle de Jong, Elisa Tjon Kon Fat, Timothy K. Connelley, Jayne C. Hope, Paul L. A. M. Corstjens, Annemieke Geluk

**Affiliations:** ^1^Department of Infectious Diseases, Leiden University Medical Center, Leiden, Netherlands; ^2^Division of Immunology, The Roslin Institute, University of Edinburgh, Easter Bush Campus, Midlothian, United Kingdom; ^3^Center for Inflammation Research, The Queen's Medical Research Institute, Edinburgh BioQuarter, Edinburgh, United Kingdom; ^4^Department of Cell and Chemical Biology, Leiden University Medical Center, Leiden, Netherlands

**Keywords:** biomarkers, bovine tuberculosis, chemokines, cytokines, diagnostics, DIVA, upconverting reporter particles, UCP-LFA

## Abstract

Bovine tuberculosis (bTB), caused by *Mycobacterium bovis* (*M. bovis*) infection in cattle, is an economically devastating chronic disease for livestock worldwide. Efficient disease control measures rely on early and accurate diagnosis using the tuberculin skin test (TST) and interferon-gamma release assays (IGRAs), followed by culling of positive animals. Compromised performance of TST and IGRA, due to BCG vaccination or co-infections with non-tuberculous mycobacteria (NTM), urges improved diagnostics. Lateral flow assays (LFAs) utilizing luminescent upconverting reporter particles (UCP) for quantitative measurement of host biomarkers present an accurate but less equipment- and labor-demanding diagnostic test platform. UCP-LFAs have proven applications for human infectious diseases. Here, we report the development of UCP-LFAs for the detection of six bovine proteins (IFN-γ, IL-2, IL-6, CCL4, CXCL9, and CXCL10), which have been described by ELISA as potential biomarkers to discriminate *M. bovis* infected from naïve and BCG-vaccinated cattle. We show that, in line with the ELISA data, the combined PPDb-induced levels of IFN-γ, IL-2, IL-6, CCL4, and CXCL9 determined by UCP-LFAs can discriminate *M. bovis* challenged animals from naïve (AUC range: 0.87–1.00) and BCG-vaccinated animals (AUC range: 0.97–1.00) in this cohort. These initial findings can be used to develop a robust and user-friendly multi-biomarker test (MBT) for bTB diagnosis.

## 1. Introduction

The members of the genus *Mycobacterium* cause major infections in humans, including tuberculosis (*Mycobacterium tuberculosis*), leprosy (*Mycobacterium leprae*), and zoonotic tuberculosis (*Mycobacterium bovis*). In cattle, *M. bovis* is the etiological agent for bovine tuberculosis (bTB) (1). Bovine TB is a globally prevalent, chronic infectious disease with dire consequences for animal trade, economics, and welfare. A very conservative estimate suggests that 50 million cattle are infected with *M. bovis*, with a loss of three billion dollars per year (2). Geographically, bTB is prevalent across the globe, with the highest prevalence in Africa and parts of Asia (3). The disease spreads among cattle herds mostly via the aerosol route and horizontal transmission via reservoir hosts (e.g., Eurasian badgers in the United Kingdom and white-tailed deer in the United States) has also been reported (4).

The antemortem diagnosis of bTB is mainly dependent on the tuberculin skin test (TST), which involves the administration of a purified protein derivative of *M. bovis* (PPDb) in the skin of the animal. Depending upon regional legislation, a comparative avian tuberculin (PPDa) is also included to exclude animals infected with only non-tuberculous mycobacteria (NTM). An increase in skin thickness is measured 72 h later, and animals are classified as reactors, inconclusive, or uninfected based on respective regional cutoffs (5). An ancillary interferon-gamma (IFN-γ) release assay (IGRA) involving *in vitro* determination of IFN-γ, the hallmark cytokine released by Th1 cells as part of the cellular immune response elicited post-mycobacterial infection, is also available commercially (6). The TST takes three days in total, is liable to operator bias, and has low specificity in the field as it is also affected by prior exposure to NTMs, co-infections (Johne's disease), and vaccination with Bacille Calmette-Guérin (BCG) (7). On the contrary, IGRAs require access to a sophisticated lab for overnight stimulation at 37°C with PPDb as well as equipment for enzyme-linked immunosorbent assay (ELISA). Moreover, IGRA outcome is also influenced by declining IFN-γ levels with advancement in the stage of the disease (8). The sensitivity and specificity ranges for TST from various international studies are between 52%−100% and 78%−99%, respectively. Similarly, for IGRA, the respective sensitivity and specificity range between 73%−100% and 85%−99% (5, 8). Regarding bovine TB disease control, some developed countries with endemic bTB rely on a test and cull strategy, whereas hardly any measures are in place in low- and middle-income countries (LMICs). BCG is the only licensed TB vaccine available for humans, but its usage in cattle is limited because it has reported limited efficacy in field trials and renders the TST ineffective as a diagnostic tool because of T cell cross-reactivity (9). To overcome the issue of cross-reactivity from PPD of different mycobacteria in BCG-vaccinated cattle (as many antigens are homologous), efforts are ongoing to develop a defined skin test from *M. bovis*-specific antigens that are absent from BCG (10).

Bovine TB is endemic in LMICs, and a major obstacle to early and accurate diagnosis is the lack of access to sophisticated laboratories. To address the challenges faced with current diagnostic tests, the goal for the future is to develop user-friendly assays that are ideally Differentiating Infected from Vaccinated Animals (DIVA) compliant. Lateral flow (LF) strip-based diagnostics, such as pregnancy and COVID-19 antigen detection tests, are commonly used due to the convenience of their use. Upconverting reporter particle (UCP)-based LF assays to detect host biomarkers have been previously applied in animals [i.e., non-human primates (NHPs) and red squirrels] utilizing assays developed for human application (11, 12). In our efforts to contribute toward improved bTB diagnostics, we report here for the first time in cattle the use of UCP-LFAs to detect host proteins and describe the associated diagnostic prospects for bTB. In contrast to our previous study on animal diagnostics based on human antibodies, this study employed antibodies of bovine origin only. The UCP technology utilizes nano-sized particles composed of rare earth lanthanides in a crystal (13). Excitation by infrared light and “upconversion” into visible light make them background-free (as this up-conversion does not happen in nature) (14). UCP-based assays have previously been shown to enable quantitative assessment of a range of analytes, e.g., hormones (15), nucleic acids (16), host serum proteins (including cytokines and antibodies) (17), pathogens (18), and potential bio-warfare agents (19). LF assays incorporating unique, sensitive UCP particles have been applied in the development of point-of-care (POC) tests for human diseases such as TB (20), leprosy (21), schistosomiasis (22, 23), and bronchiolitis (caused by Respiratory Syncytial Virus) (18). Other than for human samples, UCP-LFAs have also been pilot tested in studies conducted on samples from rodents, wild animals, and NHPs (11, 24–27). The potential of these UCP-LFAs is enormous; for example, storage of assay reagents at room temperature and the use of battery-operated (stand-alone) portable strip readers have allowed the use of these tests at several sites worldwide for the detection of human infectious diseases (20). Furthermore, the ability to multiplex, i.e., combining quantitative detection of both cellular and humoral immunity biomarkers on a single strip using a small sample volume, has also been successfully demonstrated (28).

Considering these test characteristics, we aimed to use the UCP-based LF platform to develop diagnostic tools for bTB that are sensitive and easy to use, even in low-resource areas. Identification of host protein signatures will ideally not only allow the detection of infection but also discriminate patients with various phenotypes of a disease (e.g., paucibacillary vs. multibacillary leprosy patients) (29). Using ELISAs, we previously screened a panel of nine cytokines/chemokines in both serum and supernatants of whole blood stimulated for 24 h with bovine tuberculin. The proteins included IFN-γ, Interleukin (IL) 1-β, IL-2, IL-6, IL-8, IL-17A, C-C motif ligand 4 (CCL4), C-X-C motif ligand 9 (CXCL9)/monokine induced by gamma interferon (MIG), and C-X-C motif ligand 10 (CXCL10)/interferon-gamma inducible protein-10 (IP-10). We reported that serum concentrations for all host proteins, except CXCL9 and CXCL10, were low or undetectable. However, PPDb-stimulated concentrations of IFN-γ, IL-2, CXCL10, CCL4, and CXCL9 had diagnostic potential for the detection of *M. bovis* infection and improved utility as DIVA markers (30). In this study, we report the development of UCP-LFAs for six bovine proteins identified in our previous study (IFN-γ, IL-2, IL-6, CCL4, CXCL9, and CXCL10). Considering the low levels of these host proteins in serum, we utilized PPDb-stimulated whole-blood supernatant samples for this study. Our findings show the potential of employing UCP technology for specific detection of bovine proteins for improved bTB diagnostics.

## 2. Materials and methods

### 2.1. Samples

The animal cohorts and their respective samples used in this study were described previously (30, 31). Briefly, animals were either bTB naïve (*n* = 16); *M. bovis* experimentally challenged with strain AF 2122/97 (32) (*n* = 9); BCG vaccinated (with BCG Pasteur ~1 × 10^6^ CFU/calf; *n* = 10); or BCG vaccinated and subsequently *M. bovis* challenged (*n* = 12) (30). The samples from these animals included whole blood supernatants that were stimulated for 24 h with purified protein derivatives of *M. bovis* (PPDb) at a concentration of 20 μg/ml (Veterinary Laboratory Agency, Weybridge, United Kingdom), as previously described (30, 33). The samples were taken 12 weeks after BCG vaccination or *M. bovis* challenge for the respective groups. All samples were taken under a project license from the Home Office according to ASPA guidelines and with ethical approval from local Animal Welfare and Ethical Review Boards.

### 2.2. UCP conjugates

UCP nanomaterials (200 nm NaYF4:Yb^3+^, Er^3+^ particles, functionalized with carboxyl groups) were obtained from Intelligent Material Solutions Inc. (Princeton, NJ, United States). The UCP conjugates for the six tested analytes were prepared to utilize the following antibodies: mouse anti-bovine IFN-γ (MT307; Mabtech, Stockholm, Sweden); mouse anti-bovine IL-2 (MT3B3; Mabtech, Stockholm, Sweden); chicken anti-bovine IL-6 (AHP2380B; Bio-Rad Laboratories, Hercules, CA, USA); rabbit anti-bovine CXCL9 (AHP2369B; Bio-Rad Laboratories, Hercules, CA, USA); rabbit anti-bovine CXCL10 (AHP2368B; Bio-Rad Laboratories, Hercules, CA, USA); and goat anti-bovine CCL4 (PBB0479B-050; Kingfisher Biotech, St Paul, MN, USA) at a concentration of 25 μg antibody per mg of UCP according to the methods described previously for the development of UCP-LFAs for the detection of *M. leprae* infection (21).

### 2.3. Lateral flow strips

LF strips were developed as previously described (34). Briefly, LF strips were assembled by mounting a 10-mm glass fiber sample/conjugate pad (Ahlstrom 8964), a 25-mm laminated nitrocellulose membrane (Sartorius UniSart CN95), and a 20-mm absorbent pad (Whatman Cellulose 470) on a plastic backing. The sample pad and absorbent pad each overlap 2.5 mm with the nitrocellulose, respectively, at the beginning and end. All LF strip components were obtained via Kenosha (Amstelveen, the Netherlands). Test (T) lines were sprayed using a CAMAG ATS-4 (BCON Instruments B.V., Sint-Annaland, the Netherlands) on the nitrocellulose membrane, comprising mouse anti-bovine IFN-γ (M17.1, Mabtech, Stockholm, Sweden), mouse anti-bovine IL-2 (MT11A31, Mabtech, Stockholm, Sweden), chicken anti-bovine IL-6 (AHP 2380; Bio-Rad Laboratories, Hercules, CA, United States), rabbit anti-bovine CXCL9 (AHP2369, Bio-Rad Laboratories, Hercules, CA, United States), rabbit anti-bovine CXCL10 (AHP2368, Bio-Rad Laboratories, Hercules, CA, United States), and goat anti-bovine CCL4 (PB0470B-100, Kingfisher Biotech, St Paul, MN, United States) at a concentration of 200 ng per 4-mm width. The flow control (FC) lines comprised goat anti-mouse (IFN-γ and IL-2; M8642; Sigma-Aldrich, St. Louis, MO, United States), goat anti-rabbit (CXCL9 and CXCL10; R4880; Sigma-Aldrich, St. Louis, MO, United States), rabbit anti-goat (CCL4; G4018; Sigma-Aldrich, St. Louis, MO, United States), and anti-chicken (IL-6; C2288; Sigma-Aldrich, St. Louis, MO, United States). These were sprayed at a concentration of 100 ng per 4-mm width. Ready-to-use LF strips were stored at ambient temperature in containers with silica dry pads until use.

### 2.4. UCP-LF assays

**Figure 1:**
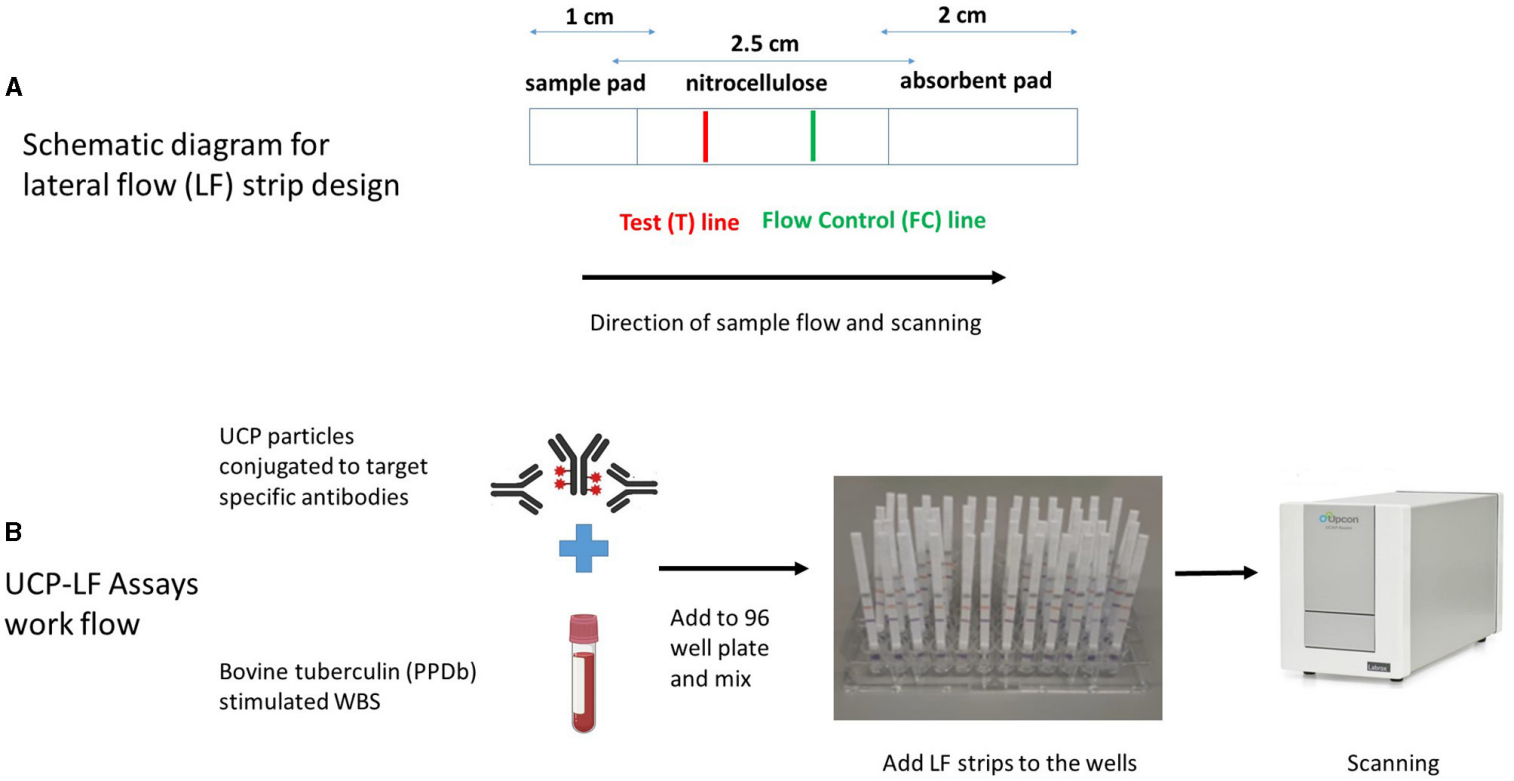
Schematic diagram of lateral flow (LF) strip design **(A)** and workflow of UCP-LFAs performed in the study **(B)**. UCP particles are first conjugated to target-specific antibodies to form the UCP conjugate. LF strips are made by stripping a test (T) line, composed of a second antibody specific for the target analyte and an isotype flow control (FC) antibody on a nitrocellulose membrane. The sample pad (the end added to the sample) and the absorbent pads are assembled to the nitrocellulose membrane on a plastic backing, and individual strips are cut and stored at room temperature. UCP-LFAs are performed by mixing a specific UCP conjugate with a sample diluted in LF buffer, followed by adding target-specific LF strips. Once immunochromatography is complete and the strips are dry, a dedicated infrared reader is used for scanning, and the signal intensity (expressed in Relative Fluorescence Units, RFUs) is calculated for both T and FC lines. Ratio (*R*) corresponds to the quantity of target analyte and is obtained by dividing signal intensities at T by the FC lines.

The LF assays were performed as previously described (35). Briefly, 200 ng of cytokine-specific UCP reporter conjugate and diluted PPDb-stimulated whole blood supernatant samples (1:10 for IFN-γ, IL-2, IL-6, CXCL9, and CXCL10, and 1:100 for CCL4) in a total assay volume of 100 μl with high salt lateral flow (HSLF) buffer (100 mM Tris pH 8.0, 270 mM NaCl, and 0.5% Tween-20) were added into 96-well plates. After 1 min of shaking at 900 rpm (PST-60HL-4; Kisker Biotech GmbH & Co KG, Steinfurt, Germany), cytokine-specific LF strips were added to the mixture in the 96-well plate, and immunochromatography was allowed to continue for at least 1 h. When dry, strips were scanned using a UCP dedicated benchtop reader (UPCON; Labrox, Finland; 980-nm infrared excitation, 550-nm emission), measuring the T and FC signals (peak area; relative fluorescent units). The results are displayed as the ratio (*R*) value of the T signal divided by the FC signals based on relative fluorescence units (RFUs). For all proteins, a dilution series ranging from 0 (i.e., buffer only) to 100,000 pg/ml was run in triplicate by spiking bovine recombinant proteins [IFN-γ, CXCL9, and CXCL10 (Bio-Rad Laboratories, Hercules, CA, United States); IL-2 and CCL4 (Kingfisher Biotech, St. Paul, MN, United States); and IL-6 (Invitrogen, Carlsbad, CA, United States)] in 100 μl of HSLF buffer. The schematic diagram for the LF strip design and the UCP-LFAs workflow are shown in Figures 1A, B.

### 2.5. Statistical analysis

Statistical analysis was performed using GraphPad Prism version 9.0 for Windows (GraphPad Software, San Diego, CA, United States; https://www.graphpad.com/). Group-wise differences were tested using the Kruskal-Wallis test and by applying Dunn's multiple comparison tests to correct for multiple comparisons. The diagnostic performance was assessed by performing a receiver operator curve (ROC) analysis and calculating the area under the curve (AUC). ROC analysis allows discrimination among groups (by plotting sensitivity against 100-specificity), followed by the calculation of optimal sensitivity and specificity cutoffs. We utilized Youden's index (defined as sensitivity + specificity– 1) for determining the optimal cutoffs, as described previously (36). The ratios (*R*) correspond to the level of the respective protein present in the sample, computed by dividing the signal intensity at the test (T) line by the signal at the flow control (FC) line. A NUM (number) score was assigned to each sample. This corresponds to the number of host proteins (out of the total six tested) that scored above the threshold of positivity based on the maximum Youden's index as previously described (28). The Pearson correlation *R*-values were calculated and expressed in the range from −1 to +1, with these extremes indicating perfectly negative or perfectly positive correlation among ratios, respectively. The statistical significance level used was *p* < 0.05.

## 3. Results

### 3.1. Development and validation of UCP-LF assays

**Figure 2:**
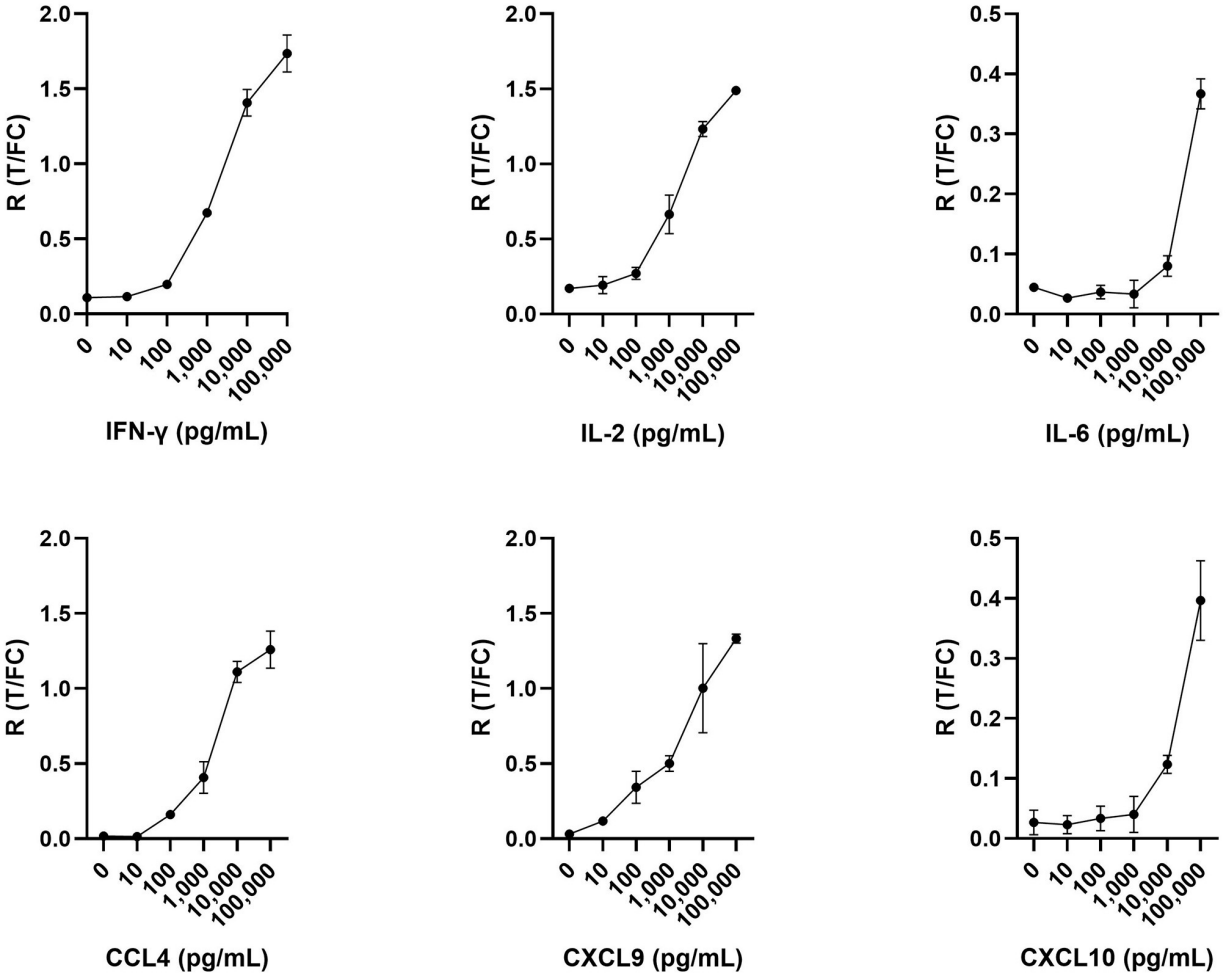
Serial dilutions in triplicate for IFN-γ, IL-2, IL-6, CCL4, CXCL9, and CXCL10. Respective recombinant bovine cytokines were spiked in assay buffer and analyzed using UCP-LFAs. Shown are the ratios (*R*) obtained by dividing the signal at the respective test (T) lines by the signal at the flow control (FC) lines for each recombinant bovine protein. Mean values with error bars (±1 SD) are shown.

UCP-LFAs were optimized using six recombinant host proteins (cytokines and chemokines). For each recombinant protein, a serial dilution (ranging from 0, i.e., assay buffer, to 100,000 pg/ml) was run in triplicate, resulting in the specific detection of all host proteins by each UCP-LFA (Figure 2). The UCP-LFAs for bovine IFN-γ, IL-2, CCL4, and CXCL9 showed high robustness, allowing specific detection between 100 and 100,000 pg/ml (the highest point in the standard curve). However, the lower limit of detection for IL-6 and CXCL10, in comparison to the other four proteins, was higher, with overall low ratios across the standard curve.

### 3.2. Differences between animal groups based on ratios determined using UCP-LFAs

**Figure 3:**
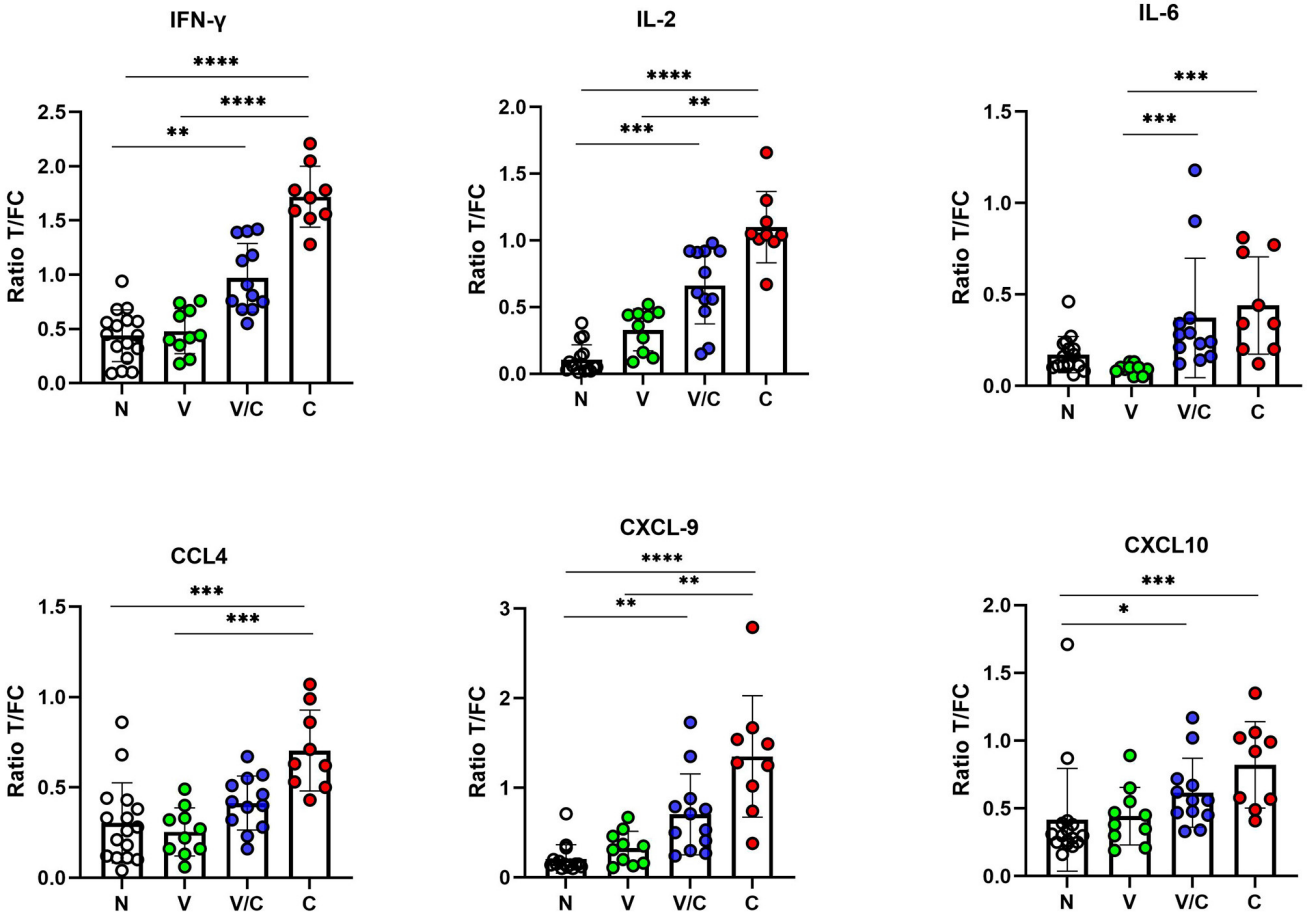
IFN-γ, IL-2, IL-6, CCL4, CXCL9, and CXCL10 levels in PPDb-stimulated whole blood supernatants were measured by UCP-LFAs. The ratios among groups were compared using the Kruskal–Wallis test with Dunn's multiple comparison post-test. N: Naive Animals (*n* = 16, empty circles); V: BCG-vaccinated animals (*n* = 10, green circles); V/C: BCG-vaccinated and *Mycobacterium bovis* challenged (*n* = 12, blue circles); C: *M. bovis* challenged only (*n* = 9, red circles). The bars of scatter dot plots show mean values, and error bars show ±1 SD. ^*^p < 0.05; ^**^p < 0.01; ^***^p < 0.001; ^****^p < 0.0001.

We evaluated individual cytokine and chemokine UCP-LFAs to discriminate between groups based on *M. bovis* infection or BCG vaccination status. Thus, the ratios obtained from PPDb-stimulated whole blood supernatants were compared (Figure 3). For discriminating *M. bovis* naïve (N) from *M. bovis* challenged (C) animals, PPDb-induced ratios for IFN-γ, IL-2, and CXCL9 showed highly significant potential as single biomarkers (*p* < 0.0001). PPDb-induced ratios for CCL4 and CXCL10 also discriminated against these groups significantly, although *p*-values were lower (0.0008 and 0.0009, respectively). BCG-vaccinated cattle (V) could be significantly differentiated from the *M. bovis*-challenged group (C) using PPDb-induced ratios of IFN-γ (*p* < 0.0001), IL-2 (*p* = 0.0053), IL-6 (*p* = 0.0001), CCL4 (*p* = 0.0007), and CXCL9 (*p* = 0.0077). This DIVA potential was not observed when PPDb-induced CXCL10 ratios were analyzed (*p* < 0.0636). None of the tested proteins could discriminate the BCG-vaccinated animals (V) from naïve animals (N). For the calves that were *M. bovis*-challenged post-BCG vaccination (V/C), differentiation was possible from the naïve group (N) using PPDb-induced ratios of IFN-γ (*p* = 0.0077), IL-2 (*p* = 0.0003), CXCL9 (*p* = 0.002), and CXCL10 (*p* = 0.02). While comparing the four study groups, only PPDb-induced IL-6 ratios could statistically discriminate this group of animals (V/C) from those that were vaccinated alone (V; *p* = 0.0002), while none of the tested proteins were found to discriminate them (V/C) from the *M. bovis*-challenged group (C) (Figure 3).

### 3.3. Receiver operator curve analysis

**Figure 4:**
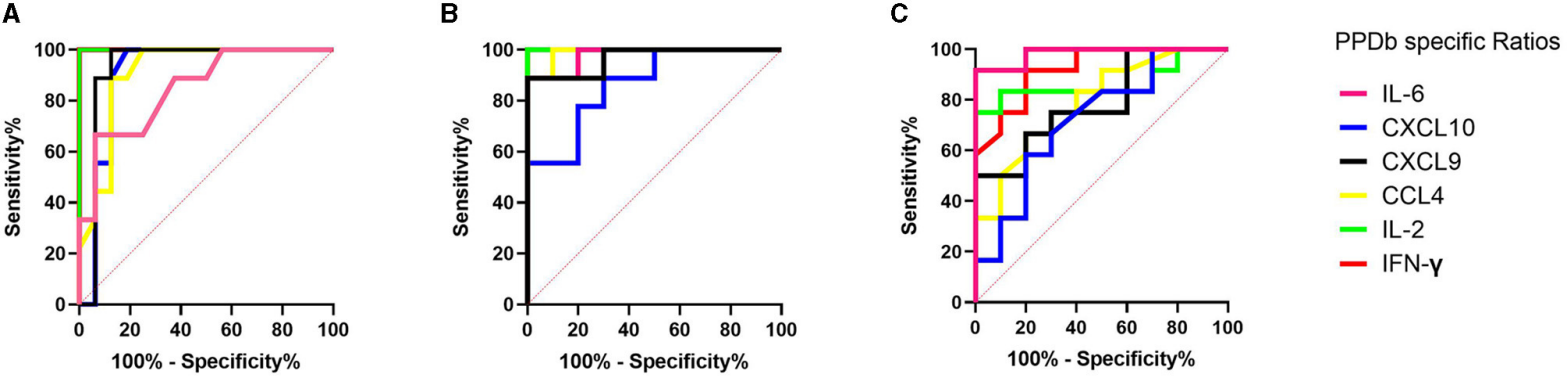
ROC curve analysis of IFN-γ (red), IL-2 (green), IL-6 (pink), CCL4 (yellow), CXCL9 (black), and CXCL10 (blue) detected in PPDb-stimulated whole blood supernatants to discriminate naïve (N) from *Mycobacterium bovis*-challenged group (C) **(A)**, BCG-vaccinated (V) from *M. bovis*-challenged group (C) **(B)**, and BCG-vaccinated group (V) from those that were *M. bovis*-challenged post-BCG (V/C) **(C)**.

A receiver operator curve (ROC) analysis was performed to determine discrimination potential among study groups (N vs. C, V vs. C, and V vs. V/C) using the ratios measured for six proteins using UCP-LFAs. The optimal cutoffs to discriminate the two groups for each host protein were determined using Youden's index (Table 1 and Figure 4).

**Table 1:** Receiver operator curve (ROC) analysis.

**Protein**	**Cutoff**	**Sensitivity (%)**	**95% CI (%)**	**Specificity (%)**	**95% CI (%)**	**AUC**	***p-*value**
**(a) Naïve vs**. ***Mycobacterium bovis*** **challenged**							
IFN-γ	*R* > 1.11	100	70.09–100	100	80.64–100	1.00	< 0.0001
IL-2	*R* > 0.53	100	70.09–100	100	80.64–100	1.00	< 0.0001
IL-6	*R* > 0.31	66.67	35.42–87.94	93.75	71.67–99.68	0.85	0.0042
CCL4	*R* > 0.47	88.89	56.50–99.43	87.50	63.98–97.78	0.91	0.0008
CXCL9	*R* > 0.37	100	70.09–100	87.50	63.98–97.78	0.93	0.0004
CXCL10	*R* > 0.40	100	70.09–100	81.25	56.99–93.41	0.91	0.0009
**(b) BCG vaccinated vs**. ***Mycobacterium bovis*** **challenged**							
IFN-γ	*R* > 1.02	100	70.09–100	100	72.25–100	1.00	0.0002
IL-2	*R* > 0.56	100	70.09–100	100	72.25–100	1.00	0.0002
IL-6	*R* > 0.17	88.89	56.50–99.43	100	72.25–100	0.98	0.0004
CCL4	*R* > 0.42	100	70.09–100	90	59.58–99.49	0.99	0.0003
CXCL9	*R* > 0.71	88.89	56.50–99.43	100	72.25–100	0.97	0.0006
CXCL10	*R* > 0.48	100	70.09–100	70	39.68–89.22	0.87	0.0071
**(c) BCG vaccinated vs. BCG vaccinated and subsequently** ***Mycobacterium bovis*** **challenged**							
IFN-γ	*R* > 0.68	91.67	64.61–99.75	80	49.02–96.45	0.92	0.0009
IL-2	*R* > 0.46	83.33	55.20–97.04	90	59.58–99.49	0.87	0.0037
IL-6	*R* > 0.14	91.67	64.61–99.75	100	72.25–100	0.98	0.0001
CCL4	*R* > 0.36	66.67	39.06–86.19	80	49.02–96.45	0.79	0.0200
CXCL9	*R* > 0.48	66.67	39.06–86.19	80	49.02–96.45	0.79	0.0200
CXCL10	*R* > 0.55	58.33	31.95–80.67	80	49.02–96.45	0.72	0.0700

IFN-γ, IL-2, IL-6, CCL4, CXCL9, and CXCL10 levels were measured by UCP-LFAs in bovine tuberculin (PPDb)-stimulated whole blood supernatants from naïve animals (N; n = 16), *M. bovis* challenged (C; n = 9), BCG vaccinated (V; n = 10), and animals that were *M. bovis* challenged after BCG vaccination (V/C; n = 12). UCP-LFA results are displayed as a ratio value (R) between test (T) and flow control (FC). The ability to distinguish naïve from *M. bovis* challenged group (a), BCG vaccinated group from *M. bovis* challenged group (b), and BCG vaccinated from BCG vaccinated and later *M. bovis* challenge group (c) was determined per protein using the ROC curve analysis and corresponding area under the curve (AUC). The study cutoff values were determined by calculating Youden's index; values above the respective cutoff for each protein were considered positive.

Ratios determined for PPDb-induced IFN-γ and IL-2 levels could discriminate *M. bovis*-challenged animals (C) from naïve animals (N) with 100% sensitivity and specificity with an AUC of 1 (*p* < 0.0001; Figure 4A). PPDb-induced ratios for both CXCL9 and CXCL10 discriminated the two groups with 100% sensitivity and specificities of 87.5 and 81.25% and AUCs of 0.93 and 0.91, respectively (*p* = 0.0004 and *p* = 0.009). For PPDb-induced CCL4, the groups could be discriminated against with high sensitivity (88.89%) and specificity (87.50%) with an AUC of 0.91 and a *p*-value of 0.0008. Finally, PPDb-induced IL-6 ratios could discriminate the groups with low sensitivity (66.67%) but high specificity (93.75%) with an AUC of 0.85 and a *p*-value of 0.0042 [Figure 4A, Table 1(a)].

ROC analysis was also performed to assess the potential of the proteins for differentiating BCG vaccinated (V) from *M. bovis*-challenged group (C). The results are shown in Figure 4B and Table 1(b). Again, PPDb-induced ratios for IFN-γ and IL-2 were most effective as single markers, discriminating these groups with 100% sensitivity and specificity with respective AUCs of 1.00 and *p*-value of 0.0002. The ratios for PPDb-induced IL-6 and CXCL9 also discriminated groups with high sensitivity (88.89%) and specificity (100%; AUC 0.98 and 0.97 and *p*-values 0.0004 and 0.0006, respectively]. A sensitivity of 100% was observed for PPDb-induced CCL4 and CXCL10 ratios with respective specificities of 90 and 70% [AUC 0.99 and 0.87, respectively, and *p*-values of 0.0003 and 0.0071; Figure 4B and Table 1(b)].

The results of the ROC analysis for discriminating BCG-vaccinated (V) individuals from those that were *M. bovis*-challenged post-BCG vaccination (V/C) are shown in Figure 4C and Table 1(c). PPDb-induced ratios for IL-6 proved most effective, i.e., sensitivity of 91.67% and specificity of 100% (AUC 0.98, *p* = 0.0001). PPDb-induced ratios for IFN-γ and IL-2 also discriminated among groups with respective high sensitivities (91.67% and 83.33%) and specificities (80% and 90%) and respective AUCs of 0.92 and 0.87 and *p*-values of 0.0009 and 0.0037. Although the specificity to discriminate these groups using CCL4, CXCL9, and CXCL10 was high (80%), the respective sensitivities were low and ranged between 58 and 67% [Figure 4C and Table 1(c)].

### 3.4. Biomarker signature

**Figure 5:**
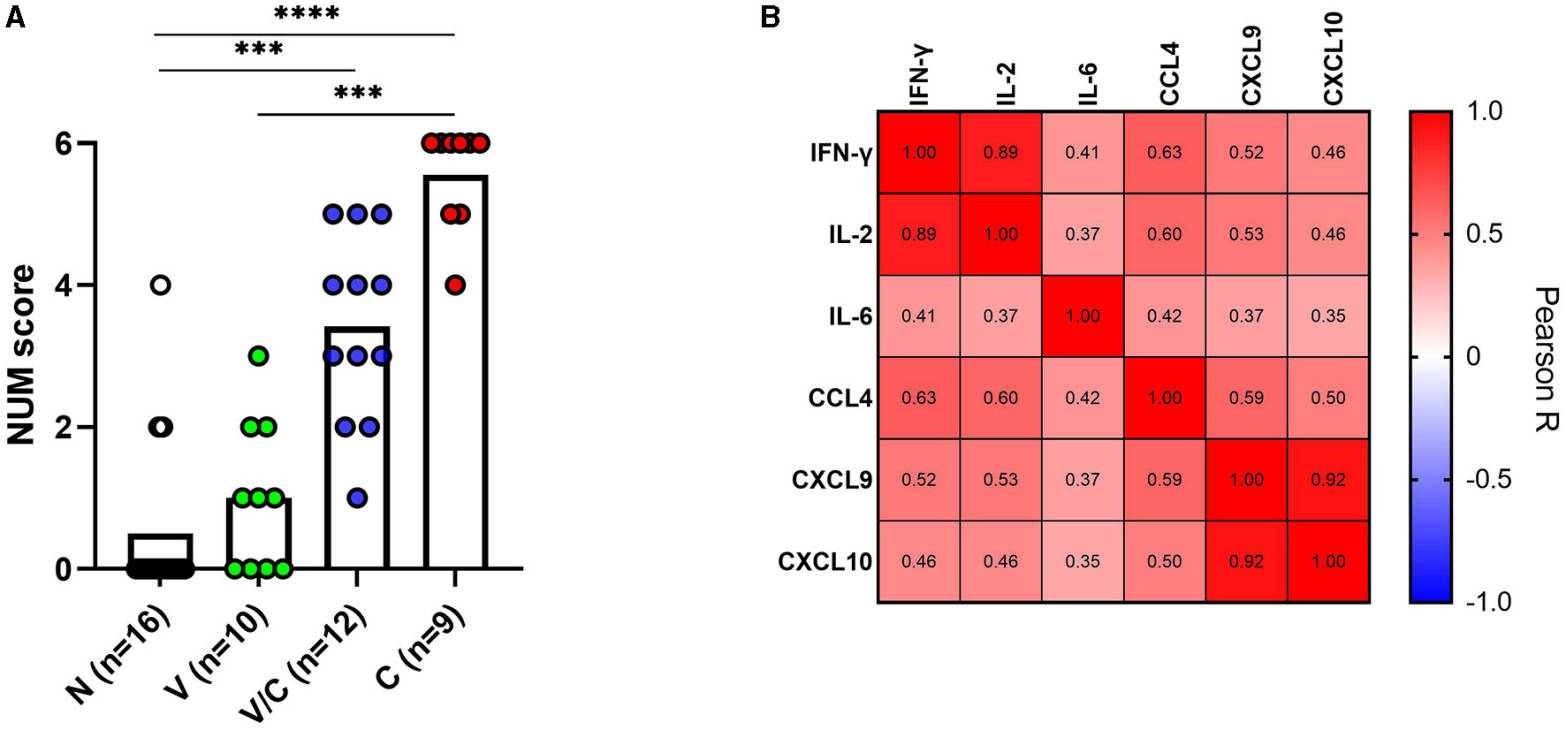
A NUM score was calculated based on IFN-γ, IL-2, IL-6, CCL4, CXCL9, and CXCL10 ratios in PPDb-stimulated whole blood supernatants for naïve (N; *n* = 16), BCG-vaccinated (V; *n* = 10), and *Mycobacterium bovis*-challenged animals with (V/C; *n* = 12) or without prior BCG vaccination (C; *n* = 9). The NUM score (*y*-axis) combines the results of six proteins, indicating the number of proteins with levels above a threshold based on the maximal Youden's index for each marker [Table 1(a)]. Group differences were determined using the Kruskal-Wallis test; the statistical significance level used was ^***^p < 0.001; ^****^p < 0.0001 **(A)**. Heatmap showing Pearson correlation among the ratios of the evaluated host proteins. The color corresponds to the Pearson *R* value as indicated in each square **(B)**.

To ascertain whether a multi-biomarker signature composed of the proteins tested in this study could provide added value to the discriminatory potential of single host proteins in overnight PPDb-stimulated whole blood supernatants, a NUM score was assigned to each sample. Here, the NUM score represents the total number out of the six proteins (IFN-γ, IL-2, IL-6, CCL-4, CXCL9, and CXCL10) detected above the marker-specific cutoff values for naïve vs. *M. bovis*-challenged group for this study cohort [as described in Table 1(a)] (28). Being a sum of the positive biomarkers per individual, NUM scores offer an easy-to-interpret and quick readout. Based on the NUM scores, naïve (N) animals could be discriminated from *M. bovis* challenged (C; *p* < 0.0001) and those that were *M. bovis* challenged post-BCG exposure (V/C; *p* < 0.0013). Similarly, we observed a significant difference between the BCG-vaccinated (V) group and the *M. bovis*-challenged (C) group (*p* < 0.0003; Figure 5A). Of 16 animals, three in the *M. bovis* naïve group yielded a NUM score of 2 or more, with all three animals showing CXCL9 and CXCL10 ratios above the respective cutoffs for this cohort. Furthermore, NUM scores varied from 1 to 5 among the vaccinated and challenged cohorts (Figure 5A).

Since IFN-γ is a currently used biomarker applied in IGRAs, we also investigated whether the ratios of IFN-γ correlated with other tested host proteins. PPDb-induced IFN-γ ratios were strongly correlated with those for PPDb-induced IL-2 [Pearson correlation *R*-value (*R* = 0.89)]; moderately with PPDb-induced CCL4 ratios (*R* = 0.63), and weakly with PPDb-induced CXCL9 (*R* = 0.52), CXCL10 (*R* = 0.46), and IL-6 ratios (*R* = 0.41; Figure 5B). Thus, a biomarker signature including other discriminatory proteins (alongside IFN-γ) that identify additional positive animals, will increase the overall diagnostic potential, especially when their mutual correlation is weak. Testing this multi-protein signature for discrimination of naïve and BCG-vaccinated groups from the *M. bovis* challenged group in the current study was not possible as the ratios for both PPDb-induced IFN-γ and IL-2 already yielded 100% sensitivity and specificity when assessed individually (Section 3.3). However, for discrimination between the BCG-vaccinated group (V) and the group that was challenged post-BCG (V/C), none of the markers yielded 100% sensitivity. Other than PPDb-induced IL-6 ratios, none of the markers showed 100% specificity. Hence, for discrimination between these two groups, a six-biomarker signature with a cutoff value of ≥3 provided 75% sensitivity and 90% specificity (Table 2). Biomarkers were then individually deleted from this 6-marker signature based on their relative AUC, i.e., the marker with the lowest AUC was removed first until we were left with the best-performing markers. The results indicate that PPDb-induced IL-6 ratios performed best individually (91.67% sensitivity, 100% specificity, and AUC 0.98), and for this particular sample set, no added value (improvement in sensitivity and specificity) was observed by combining other markers with PPDb-induced IL-6 (Table 2).

**Table 2:** NUM scores for biomarker signature.

**Biomarker(s) in NUM score**	**AUC**	**Cutoff**	**Sn/Sp (%)**
IL-6	0.98	=1	91.67/100
IL-6, IFNγ	0.83	≥1	66.67/100
IL-6, IFNγ, IL-2	0.93	≥1	91.67/90
IL-6, IFNγ, IL-2, CCL4	0.96	≥1	100/80
IL-6, IFNγ, IL-2, CCL4, CXCL9	0.94	≥2	83.33/90
IL-6, IFNγ, IL-2, CCL4, CXCL9, CXCL10	0.92	≥3	75/90

Biomarker signature for discrimination between BCG-vaccinated (V) and BCG-vaccinated and later *Mycobacterium bovis*-challenged (V/C) groups. Individually, IL-6 ratios from PPDb-stimulated whole blood supernatants gave the best discrimination among these groups [Table 1(c)]. A cutoff for positivity for each marker was determined using the maximal Youden's index. Using these cutoffs, a NUM score was calculated for each animal referring to the number of biomarkers that scored above the positivity threshold. AUC, the area under the curve; Sn/Sp sensitivity/specificity.

## 4. Discussion

The development of user-friendly diagnostics for bTB can have multiple benefits in resource-constrained settings. These could be employed to reduce transmission within cattle and improve the detection of zoonotic TB in humans, thus contributing to both improved animal and public health. The use of TST and ancillary IGRA with stringent slaughter measures has proven successful in decreasing the disease burden in developed countries (9). However, the economic impact of such control measures would be enormous for all the involved stakeholders in LMICs. The development of host biomarker signature-based tests that can ideally be DIVA (enabling strategic vaccination with BCG) could therefore be a promising way forward.

In this proof-of-concept study, we report the development of the first UCP-LFAs for the detection of bovine host proteins. The bovine proteins studied were selected for UCP-LFA development as they exhibited (combined) potential to discriminate naïve from *M. bovis*-challenged animals and/or were shown to be DIVA-compliant biomarkers in our previous study using individual ELISAs (30). Results generated by user-friendly UCP-LFAs similarly differentiate *M. bovis* infection and/or BCG vaccination status. Currently, ELISA is an established diagnostic technique, but the disadvantages associated with time, labor, cost, and experienced personnel demand investigation into more efficient alternatives (37). The LFA format presented here provides an attractive alternative, as targeted markers can be detected by a simple approach using a relatively small sample volume. Although this study utilized a benchtop reader to quantify results, in the field, the same UCP-LFA strips can be analyzed using hand-held, battery-operated portable readers, as has previously been shown for TB and leprosy diagnostics (20, 28, 34, 38).

The data reported herein showed maximal differential potential among the three study groups using individual proteins (PPDb-induced IFN-γ, IL-2, and IL-6), and no additive benefit for diagnostic improvement was observed when proteins were combined for a biomarker signature. Although IFN-γ alone provided 100% sensitivity and specificity in discriminating naïve and BCG-vaccinated animals from *M. bovis*-challenged animals, it is released in much lower concentrations following stimulation with PPDb compared to proteins such as IL-2, CCL4, and CXCL10 (30). Moreover, in contrast to naturally infected animals, stronger immune responses are usually observed in experimentally infected animals, such as those that were studied here (33, 39). Similarly, in anergic and chronically infected animals, in which IFN-γ responses may no longer be present, the quest for additional biomarkers becomes even more significant. Various bTB studies have strongly argued in favor of the inclusion of biomarkers other than IFN-γ in a host protein signature/multiple read-out system for improving diagnostic sensitivity and specificity (in comparison to using either TST or IGRA alone or in parallel) (40–42). Building on these initial findings, we can discover and validate an optimal biomarker signature based on regional control aims, i.e., a DIVA-compliant user-friendly assay allowing BCG vaccination and/or allowing accurate diagnosis with no false positives or negatives for eradication schemes in high-income countries. Other studies reported that CXCL10 is an extremely important diagnostic marker and can be detected in much higher concentrations compared to IFN-γ (30, 43). Therefore, the measurement of CXCL10 might thus function as an alternative or adjunct tool to IGRA. Only PPDb-induced IL-6 ratios showed statistical discriminatory potential between BCG-vaccinated animals and those that were challenged with *M. bovis* post-BCG while comparing the four animal groups in the study. It is therefore worthwhile to improve the sensitivity of assays for IL-6 and CXCL10. Two animals in the naïve group had a NUM score of 2, and one animal had a NUM score of 4. When ratios from their medium-only stimulated samples were tested, they were found to be equal to or even higher than the respective PPDb-induced ratios (data not shown). This is consistent with other studies, which reported that while levels of these chemokines are mostly higher in infected animals in response to PPDb stimulation compared to medium/controls, this might not always be the case (44, 45). While exact mechanisms are unclear, it is likely that the elevated levels observed in unstimulated or serum samples are due to these chemokines being non-specific pro-inflammatory markers produced *in vivo* before sampling (43, 46). Lower levels in PPD-stimulated samples in comparison to unstimulated samples may sometimes be due to the release of inhibitory cytokines such as IL-10 in response to the activation of antigen-specific memory lymphocytes (45). Despite this, when a NUM score cutoff of 3 is considered the cutoff for *M. bovis* infection, all *M. bovis* challenged and naïve animals (except for the one with high ratios for medium stimulation) from this cohort were correctly classified while showing a range of positive and negative results among the vaccinated and challenged cohort NUM scores (1–5). The latter is expected and indicates the amply described partial protection conferred by BCG after a virulent challenge (31, 33, 47).

To the best of our knowledge, this is the first study describing the utility of the quantitative UCP reporter technology utilizing bovine antibodies for bTB diagnostics. Qualitative lateral flow-based approaches for bTB have been tested by other groups: Alonso et al. recently described a gold nanoparticle-based test to detect humoral responses against MPB83. They showed the potential of the platform to detect animals that were TST-negative but had confirmed *M. bovis* infection (based on PCR positivity from lesions at post-mortem) and hence could complement the existing CMI-based tests (TST and IGRA) (48). Conflicting initial findings have been reported when lipoarabinomannan (LAM) antigen tests and Lionex Animal TB antibody tests (comprised of non-disclosed proprietary *M. bovis*-specific antigens) have been assessed in various bTB settings. In one study carried out on Ethiopian cattle, while the specificities of both tests were excellent (98%), the sensitivities were low, i.e., 72 and 54%, respectively (49). The findings of Kelley et al., however, did not support the usefulness of these POC tests for bTB diagnostics (50). Similar to the study reported herein, limited sample sizes were used, and each of the potential rapid test platforms requires additional assessment and validation on larger cohorts of animals.

Further research using larger animal cohorts of experimentally and naturally *M. bovis*-infected animals is needed to validate current findings. It will also be key to account for co-infections with related mycobacteria, particularly *Mycobacterium avium paratuberculosis* (MAP), the causative agent of Johne's disease, which has been reported to alter the bias of the immune response and cytokine expression (51). Furthermore, the assays were currently developed only for six cytokines/chemokines identified in our previous study (30). In mycobacterial diseases, the cellular immune responses tend to decrease as the disease progresses and bacteria multiply, leading instead to increased humoral responses (35, 52). Sridhara et al. (53) recently reported enhanced diagnostic sensitivity by complementing TST and IGRA with the detection of antibodies against the MPB70/MPB83 fusion protein (determined by Dual Path Platform technology) in sample cohorts from the United States and Spain. Future studies could involve testing *M. bovis-*specific antigens on the UCP platform. The inclusion of a humoral marker along with cytokines/chemokines on a single MBT platform [as shown for the leprosy prototype (28)] could potentially be useful to detect *M. bovis* across the disease spectrum. Finally, the levels in serum for most of the evaluated proteins obtained with UCP-LFAs were low or undetectable (data not shown) (30). Future studies should focus on the discovery, development, and optimization of UCP-based assays for promising biomarkers detectable in serum (54, 55). This can be more useful for developing accessible diagnostics with easier field applicability. With regard to the challenges that field settings pose for blood incubation steps in bTB diagnostics, future studies should exploit the advances in simulation platforms. Simultaneous detection of IFN-γ and IP-10 from plasma stimulated in QuantiFERON^®^ Gold tubes (pre-coated with ESAT6, CFP10, and TB-7.7 antigenic peptides) identified all *M. bovis* culture-positive African buffaloes compared to using TST and IGRA alone or in parallel (56). Similarly, another stimulation platform composed of polyester beads engineered to express *M. tuberculosis*-specific ESAT6, CFP-10, and MTB 7.7 has been developed (57). Beads enhanced cytokine release assay (BECRA) was subsequently tested and indicated the diagnostic promise of IFN-γ, IL-2, IP-10, and CCL11, as single markers and also in various combinations, for discriminating smear and/or culture-positive human pulmonary TB patients from both TST-positive and TST-negative healthy individuals (57). Initiating stimulation directly in these field-friendly tubes will lead to a reduction in total assay time. Furthermore, using a multi-biomarker, lateral flow detection format (28) as a follow-up to these innovative platforms presents huge opportunities and can advance the eventual goal of developing globally applicable bTB diagnostics.

## 5. Conclusion

We show for the first time that the development of UCP-LFAs for the detection of bovine proteins is feasible and demonstrates good performance. While this is a proof of principle, this format allows rapid and less complex analysis of the protein levels in PPDb-stimulated samples, and therefore its potential for being a user-friendly alternative to lab-based ELISAs requires further evaluation and validation. To achieve the ambitious targets set to eliminate bTB, accurate diagnosis of *M. bovis*, which has largely been neglected, has to be prioritized higher in healthcare systems (58). The development of sensitive and specific assays as described here will allow improved diagnostics for bovine tuberculosis.

## Data availability statement

The original contributions presented in the study are included in the article/supplementary material, further inquiries can be directed to the corresponding author.

## Ethics statement

All samples were taken under Project License from the UK Home Office according to ASPA guidelines and with ethical approval from local Animal Welfare and Ethical Review Boards. The study was conducted in accordance with the local legislation and institutional requirements. No potentially identifiable images or data are presented in this study.

## Author contributions

Conception, design, and funding: AG, JH, TC, and PC. Design of UCP-LFA strips: LP, PC, AvH, and AG. Production of and experiments with UCP-LF strips: HK, LP, ZZ, ET, and DdJ. Wrote first draft: HK. Performed statistical analysis: HK, LP, and AvH. Writing—review and editing: LP, TC, AvH, JH, PC, and AG. All authors contributed to the manuscript revision, read, and approved the submitted version.
